# Insights Into Pediatric Secretory Carcinoma of the Salivary Gland: A Case Report

**DOI:** 10.7759/cureus.60355

**Published:** 2024-05-15

**Authors:** Guillermo J Serrano-Meneses, Sofia Brenes Guzmán, Martín A Serrano-Meneses, Alberto Delgado-Porras

**Affiliations:** 1 Pediatric and Neonatal Surgery, Hospital Infantil Privado, Star Médica, Mexico City, MEX; 2 Pediatric Surgical Oncology, Hospital Infantil Privado, Star Médica, Mexico City, MEX; 3 Chemical and Biological Sciences, Universidad de las Américas Puebla, Puebla, MEX

**Keywords:** without chemotherapy, malignant tumor resection, surgery, mammary analog secretory carcinoma, malignant salivary gland neoplasm

## Abstract

Secretory carcinoma of the salivary gland (SCSG) is a rare head and neck tumor in adults and exceptional at the pediatric age. Its varied histological subtypes and distinct clinical presentation pose diagnostic and therapeutic challenges. Therefore, standardized guidelines are of utmost importance for the care of these patients, especially in children. Here we present an 11-year-old male presented with a left cheek mass initially diagnosed as lipoma. A wide resection was performed and SCSG was revealed in the histopathologic and immunohistochemistry analysis. The presentation of this case provides valuable information on the diagnostic and therapeutic complexities of SCSG. It emphasizes the need for standardized guidelines and further research to optimize pediatric patient outcomes. Overall, this case report is a crucial resource for clinicians and researchers, highlighting the importance of interdisciplinary collaboration and early intervention in managing SCSG.

## Introduction

Secretory carcinoma of the salivary gland (SCSG) represents a rare and heterogeneous group of tumors, comprising less than 10% of all pediatric head and neck neoplasms. SCSG poses a significant diagnostic and therapeutic challenge due to its diverse histological subtypes, variable clinical presentation, and lack of standardized guidelines for pediatric management, especially in small biopsy and cytology samples [1,2]. This review provides a comprehensive overview of SCSG, which involved its epidemiology, clinical features, diagnostic modalities, histopathological characteristics, molecular insights, treatment strategies, and its prognosis. By synthesizing current evidence and clinical expertise, this review aims to enhance the understanding and facilitate optimal management of SCSG in pediatric patients.

## Case presentation

**Figure 1:**
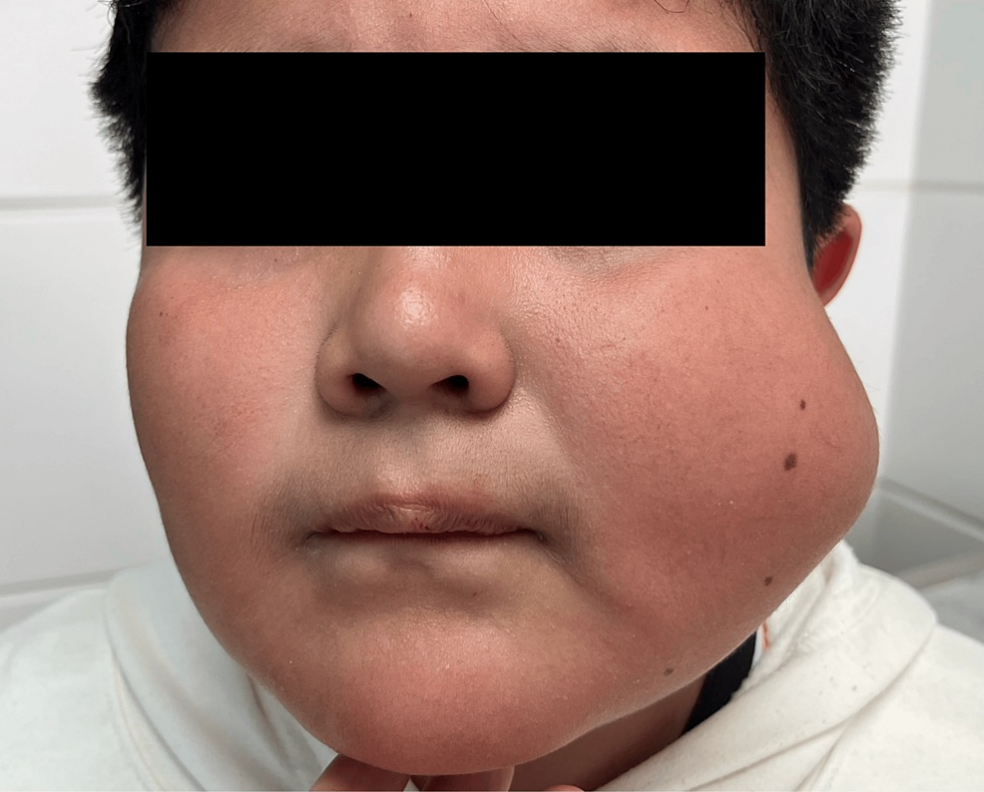
Clinical picture of the patient A 6-centimeter lesion is well-defined and firm on the left cheek.

**Figure 2:**
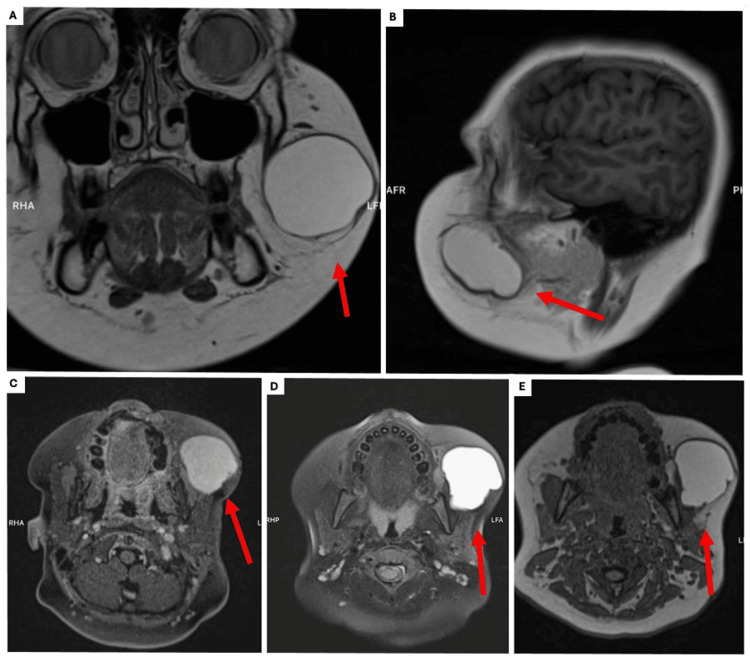
Magnetic Resonance Imaging (A) An oval, well-defined lesion with a thin wall was found in the left maxillary soft tissue on the coronal view on T1. (B) T1 on the sagittal view. (C) T1 on axial view. (D) The same lesion was hyperintense also on T2. (E) No enhancement with contrast and no infiltration of the surrounding tissue.

An 11-year-old male with a maternal family history of gastric, lung, and breast cancer was referred for a firm, but not indurated left cheek tumor which appeared 24 months before our first evaluation. In December 2021, a first failed attempt of complete resection was performed by the first doctor who evaluated him, unfortunately, we did not have access to the clinical record, but only to the histopathology report that initially confirmed a lipoma. In June 2022, volume growth resumed gradually to approximately 4 cm with ultrasound revealing a cystic lesion with a well-defined capsule and an isoechoic image inside, without evidence of involvement of surrounding tissues. It was not until October 2023 that a second medical evaluation resulted in a tumor puncture with a decrease in size and the extraction of dark blood. Unfortunately, no samples were sent for histopathological study. A week after the puncture there was a significant tumor growth, even larger than the initial size, therefore, the patient was referred to our pediatric surgery consultation (Figure 1). A magnetic resonance revealed an oval, well-defined lesion with a thin wall in the left maxillary soft tissue of 54 x 51 x 48 mm. It was hyperintense on T1 and T2 with no enhancement with contrast and no infiltration of the surrounding tissue (Figure 2).

**Figure 3:**
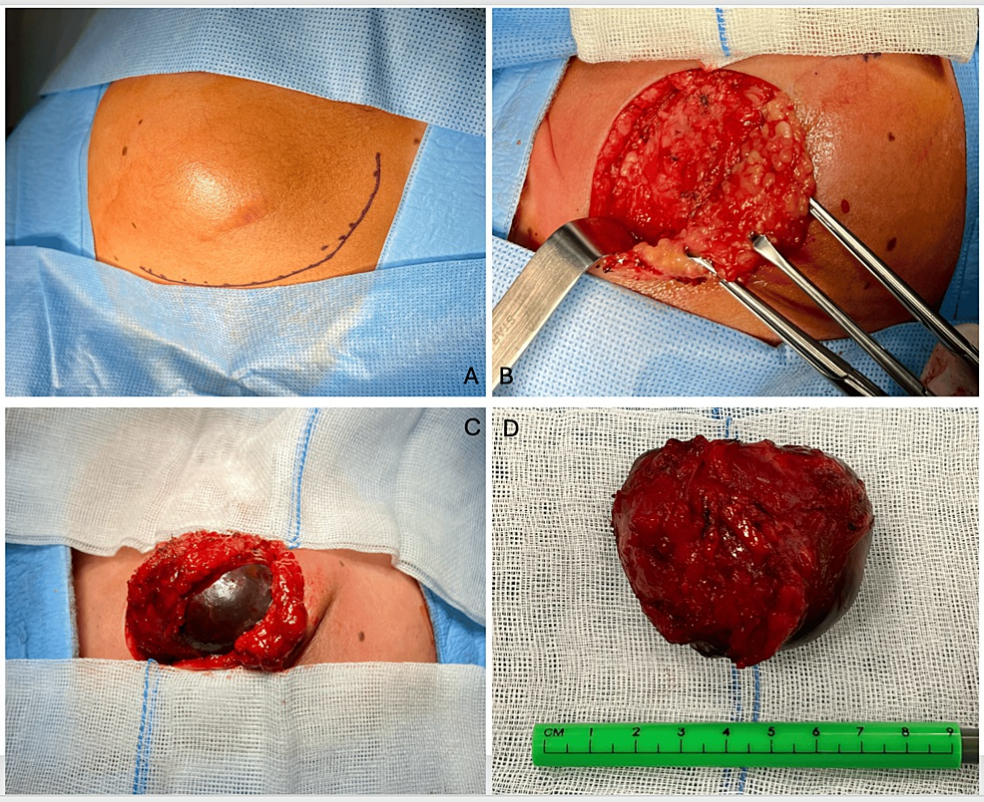
Surgery (A) An incision is marked on the patient's skin on the left mandibular line 1.5 centimeters away from the mandibular branch, with a length twice that of the lesion, extending longitudinally on both sides of the lesion to prevent rupture. (B) Dissection of the superficial tissues (subcutaneous tissue). (C) Dissection of the tumor on the periphery of the tumor. (D) 6-centimeter diameter tumor extracted with free margins of tumor macroscopically.

In January 2024, a wide resection of the lesion was performed by our surgical team. A 5-cm diameter tumor with cystic characteristics and evidence of old bleeding inside was observed; the tumor was fully removed (Figure 3). The sample was sent for histopathological study with the following findings: a predominantly cystic tumor, well-circumscribed and encapsulated. Intracystic cellular proliferation of monotonous cells with eosinophilic cytoplasm, round nuclei, fine chromatin, and occasional pinpoint nucleoli was observed. Tubular structures and microcysts with eosinophilic colloid-like material that is positive with Periodic Acid Schiff (PAS) staining were present. The cyst wall was fibrous, with moderate multifocal chronic inflammation. Immunohistochemically, the tumor cells express broad-spectrum cytokeratin (AE1-3), cytokeratin 7, GATA3, and Protein S100; they were negative to Actin and P63. The Ki67 was 20%. Outside the cystic wall, adipose tissue and a few serous salivary acini were observed. The lesion was completely excised with clean margins. The histological appearance and immunophenotype were consistent with the diagnosis of a pediatric secretory carcinoma of the salivary gland, cystic type. It was considered a low-grade lesion, but a long-term follow-up was strongly advised by the pathologist. As it is mandatory, we requested an evaluation by the pediatric oncology team for a long-term follow-up. One month after the surgery, a PET/CT scan was performed, and no hypermetabolic lesions that suggest tumoral activity were identified, then, chemotherapy and radiation therapy were not considered necessary.

## Discussion

SCSG represents a rare and heterogeneous group of tumors comprising less than 10% of all pediatric head and neck tumors with an incidence of 0.8/1,000,000. They tend to appear in the second decade of life [3,4], and are also more frequently malignant in children with a poorer prognosis (50%) than adults (10-25%). The lack of specific pediatric standardized guidelines makes diagnosis and treatment of these tumors challenging for pathologists, surgeons, and pediatric oncologists [4-7], hence reporting this clinical case might facilitate detection of similar or identical cases.

SCSG affects mainly parotid and submandibular glands. Minor salivary gland tumors are uncommon but comprise the second most common site in children [8]. Mucoepidermoid carcinoma (MEC) accounts for 50% of all pediatric salivary gland carcinomas, with acinic cell carcinoma following at 25-35%. Both MEC and acinic cell carcinoma are typically classified as low-grade tumors [6]. In rare cases, adenocarcinoma, and adenoid cystic carcinoma, which are known for their more aggressive behavior, may also be present. Metastases at the time of diagnosis are uncommon, with the lungs being the most common site, followed by liver and bone tissues [6,9,10]. There is not a genetic predisposition syndrome, but familial clustering has been reported. The etiology is related to multiple susceptibility genes and environmental factors [6, 11]. An Epstein-Barr virus latent infection identified by in situ hybridization has been correlated with the appearance of these tumors [12].

Usually, patients present as slow-growing, asymptomatic, fixed, painless nodules commonly incidentally identified, with an average of 2 to 5 cm on examination; they often delay attention for 12-24 months [13]. The clinical assessment should encompass a comprehensive examination of the cervical lymph nodes and a neurological evaluation [6,9]. In the initial diagnostic approach, the first imaging method is the cervical ultrasound with Doppler, which can provide an overall tumor characteristic. Magnetic resonance imaging (MRI) of the head and neck is essential when a neoplasm is suspected, as it confirms the exact location of the tumor and its spread in the surrounding area. This imaging technique offers crucial insights into lymph node and bone tissue involvement, aiding in the initial assessment of the possible causes of the tumor [6]. Computed tomography (CT) can be performed when MRI is not accessible or feasible [14-16]. The effectiveness of fluorodeoxyglucose (FDG) positron emission tomography (PET/CT) remains unclear because the sufficient accuracy to discriminate benign from malignant lesions is yet to be determined [6]. However, it is certainly useful for detecting lymph nodes and metastases [15,17,18-20].

Fine needle aspiration or ultrasound-guided biopsy can be helpful in the setting of an unresectable tumor before attempting to perform any mutilating surgery, or when there is a reasonable doubt about the definitive diagnosis after the first histological examination [21]. However, this task is complex not only because of the similarities in histological and immunohistochemical characteristics with normal salivary gland elements but also due to the specialized expertise needed to differentiate between benign and malignant salivary gland secretions [22,23]. The MRI characteristics described, which are consistent with salivary gland carcinomas (SGCs) and noted in our patient, include (1) Shape: Typically, round with well-defined borders. (2) Signal Intensities: Predominantly long or mixed long and short signals on T1 or T2 images, along with high signals on fat suppression sequences. (3) Diffusion Characteristics: Minimal high b-value diffusion with high signals and varying degrees of uneven enhancement, ranging from slight to significant [24]. A few tumors have cysts likewise our patient. It is common for these tumors to display a lobulated growth pattern with fibrous septa containing microcystic/solid components, along with tubular, follicular, and papillary cystic structures that feature unique luminal secretions [13,24,25].

Histology plays a crucial role in confirming the diagnosis. Under high magnification, the tumor cells typically appear round or oval with mild atypia. They exhibit eosinophilic granular or vacuolated cytoplasm and have small, uniform nuclei [24,26,27]. Cytology can assist in differentiating SGCs from non-neoplastic conditions as well as benign or malignant tumors that are not of epithelial origin in the salivary gland area [6,28]. The secretions typically show positive staining for periodic acid Schiff (PAS) and diastase-resistant material, along with positive staining for markers such as S-100, mammaglobin, vimentin, and cytokeratin-19. Moreover, molecular detection through fluorescent in situ hybridization (FISH) is considered the gold standard for identifying the frequently encountered ETV6-NTRK3 fusion gene, associated with the translocation t (12;15) (p13; q25). This fusion gene is unique to this type of tumor and has not been reported in any other salivary gland tumors. The ETV6‑NTRK3 fusion gene encodes a tyrosine kinase that promotes oncogenesis by causing increased cell proliferation and perpetuates tumor cells [29-32]. Certain molecular disorders in malignant SGCs have been identified recently, therefore, to confirm the diagnosis in morphologically ambiguous tumors, molecular testing delivers crucial information that benefits patients [6,33,34].

The mainstay of surgical treatment is the complete resection with clear margins of the tumor and the preservation of both, motor and sensitive function. It is also important to minimize the likelihood of tumor spreading by avoiding partial resection or excision biopsy. In the close or positive postsurgical margins scenario an additional surgical procedure must be carried out to ensure microscopically complete resection without mutilation, however, to achieve clear surgical margins can be challenging in some patients [35]. Surgery modalities depend on the tumor’s variant, location, and extent. The criteria for performing cervical lymph node dissection, a second look procedure, adjuvant radiotherapy (RT), and chemotherapy still require a clear definition [6,17,36]. Apart from facial nerve paralysis, common complications following surgery include Frey syndrome, issues with scarring, sialocele formation, bleeding, hematoma, fistula formation, and first bite syndrome [6,37]. The use of adjuvant radiotherapy in pediatric SGCs and adjuvant chemotherapy lacks substantial evidence to endorse their routine use, typically being reserved for palliative care in cases of recurrent or metastatic disease [6, 38].

The prognosis of salivary gland secretory carcinoma (SGSC) is not solely reliant on the success of the initial surgical procedure [24,39,40] but is also influenced by factors such as age, clinical stage, and the Ki-67 proliferation index. These factors collectively contribute to the overall prognosis and treatment outcomes for individuals diagnosed with this type of carcinoma [24,41]. Moreover, prognostic stratification is a significant challenge due to the exceptional rarity of these tumors [42]. The overall prognosis for primary SGCs in children and adolescents is considered favorable, boasting a 90% overall survival rate at the 10-year mark [6]. Recurrence varies considerably depending mainly on the histologic type and tumor stage [43]. Surveillance with a strict follow-up for at least five years is highly recommended and should focus on recurrence and potential long-term chemotherapy, radiotherapy, and surgical side effects, even after several years [44].

## Conclusions

The impact of this case extends beyond individual patient care and may enhance the collective knowledge base of clinicians and researchers. The detailed discussion in this text highlights the rarity and complexity of SCSG, and emphasizes the need for standardized guidelines and further research to improve outcomes for pediatric patients with this rare and heterogeneous group of tumors.

Overall, this text serves as a valuable resource for clinicians and researchers involved in the care of pediatric patients with SCSG. It underscores the importance of interdisciplinary collaboration, early recognition, and appropriate intervention in optimizing patient outcomes. Moving forward, continued research efforts and the development of standardized guidelines are essential to address the diagnostic and therapeutic challenges posed by SCSG and ultimately improve patient care.
